# Total colonic aganglionosis: a bibliometric analysis of trends and themes (1978–2024)

**DOI:** 10.1186/s13023-026-04347-w

**Published:** 2026-05-16

**Authors:** Jing Sun, Xiaoli Xie, Fei Liu, Xiaogang Xu, Menglong Lan, Huimin Chen, Cuiping Liang

**Affiliations:** 1 Department of Clinical Nutrition, Guangzhou Women and Children’s Medical Center, Guangzhou Medical University, 9 Jinsui Road, Guangzhou, Guangdong 510623 China; 2 Department of Surgical Neonatal Intensive Care Unit, Guangzhou Women and Children’s Medical Center, Guangzhou Medical University, 9 Jinsui Road, Guangzhou, Guangdong 510623 China; 3 Department of Pediatric Surgery, Guangzhou Women and Children’s Medical Center, Guangzhou Medical University, 9 Jinsui Road, Guangzhou, Guangdong 510623 China; 4 Department of Gastrointestinal Surgery, Guangzhou Women and Children’s Medical Center, Guangzhou Medical University, 9 Jinsui Road, Guangzhou, Guangdong 510623 China

**Keywords:** Total Colonic Aganglionosis, Pediatric Gastroenterology, Bibliometric analysis, VOSviewer, CiteSpace

## Abstract

**Background:**

Total colonic aganglionosis (TCA) is a rare but severe congenital disorder that impacts the colon’s ability to function properly due to the absence of ganglion cells. Investigating research trends in the diagnosis and treatment of TCA can offer valuable insights into advancements in clinical practices, surgical techniques, and future directions in pediatric gastroenterology.

**Methods:**

Literatures related TCA were explored through a search in the Web of Science Core Collection (WoSCC) database, spanning the period from 1978 to 2024. Bibliometric analysis and data visualization were conducted using VOSviewer, CiteSpace, and the R package “bibliometrix.”

**Results:**

A total of 281 articles were analyzed, accumulating over 4,010 citations. The USA led in productivity, with Paris City University as the top institution, and *Journal of Pediatric Surgery* emerged as the leading journal. Prem, Puri was identified as the most influential author. Current hotspots included genetic mutations, management, clinical guidelines, diagnosis, and pull-through for TCA. Keywords burst analysis showed emerging interests in “diagnosis” (2020–2024), “management” (2022–2024), and “pull-through” (2022–2024).

**Conclusion:**

This study presents a bibliometric analysis of TCA, focusing on emerging research trends, influential publications, and global collaborations. The findings identify key advancements in the understanding and treatment of TCA, providing valuable insights for both clinical and basic research in pediatric gastroenterology.

**Clinical trial number:**

Not applicable.

**Supplementary Information:**

The online version contains supplementary material available at 10.1186/s13023-026-04347-w.

## Background

Total Colonic Aganglionosis (TCA) is a rare congenital disorder, affecting less than 10% of all Hirschsprung disease (HD) cases [1]. It is characterized by the absence of ganglion cells across the entire colon, resulting in severe intestinal obstruction in neonates and infants [1, 2]. The pathogenesis of TCA remains incompletely understood; however, aberrant neural crest cell migration and mutations in the RET gene are strongly implicated [3]. Unlike HD, which may occasionally present in adults, TCA occurs almost exclusively in the pediatric population and manifests as more severe obstructive symptoms [4]. Clinical features typically emerge shortly after birth and may include delayed meconium passage, abdominal distension, vomiting, and feeding difficulties [5]. If not diagnosed and treated promptly, TCA can lead to life-threatening complications such as Hirschsprung-associated enterocolitis (HAEC), intestinal failure, or related genetic syndromes, with an overall mortality rate ranging from 2% to 20% [6–8]. Surgical intervention, involving resection of the aganglionic segment and bowel reconstruction, remains the cornerstone of treatment. Despite improvements in surgical techniques and perioperative care, postoperative complications, such as persistent constipation, diarrhea, malnutrition, and enteritis, are common, underscoring the need for long-term multidisciplinary management [9, 10]. Additionally, due to the lack of long-term follow‐up, no reconstructive surgical technique has emerged as superior to others with regard to functional outcomes or complications among J pouch with ileoanal anastomosis (JIAA), straight ileoanal anastomosis (SIAA) and the Duhamel operation [6]. Thus, TCA continues to pose significant challenges in both surgical management and overall patient care.

Despite progress in TCA research, no comprehensive study has summarized current trends and future directions. Bibliometric analysis helps identify key research areas and collaborations, guiding strategic decisions. While prior bibliometric study has focused on HD, TCA-specific research remains underexplored [11]. This analysis aims to provide a comprehensive overview of TCA research, highlighting key institutions, scholars, historical development, research hotspots, and future directions, offering valuable insights for both clinical and basic research.

## Methods

### Search strategies and data collection

A literature search on TCA was conducted using the Web of Science Core Collection (WoSCC), a comprehensive database covering publications from 1978 to 2024. Only English-language publications were included, and document types were restricted to original research articles to focus on primary scientific contributions. To avoid database renewal-related deviations, we performed the literature retrieval on a single day (Nov 13, 2024). The search strategy was as follows: TS = (“total colonic aganglionosis” OR “total intestinal aganglionosis” OR “total colonic Hirschsprung disease” OR “entire colon aganglionosis” OR “total colonic megacolon”). On November 13, 2024, we conducted comprehensive literature searches and compiled all retrieved records into plain text format within the same day to ensure data consistency and prevent temporal discrepancies in collection. These consolidated source files were prepared for compatibility with multiple bibliometric analysis tools. Using the “export” function, we downloaded data from WoSCC database, including titles, authors, keywords, abstracts, institutions, countries, languages, and cited references, with the “record content” option set to “full record and cited references” to minimize errors and support reproducibility in subsequent analyses.

### Statistical analysis

Microsoft Excel 2019, R “bibliometrix,” VOSviewer 1.6.20, and CiteSpace 6.3.R1 were utilized for data analysis and visualization. Excel was used for annual publication trends, citation frequencies, journal impact factors, and institutional contributions [12]. The R “bibliometrix” package enabled publication output visualization and institutional article counts [12]. VOSviewer facilitated network visualizations for author, institution, and country collaborations, as well as keyword co-occurrence analysis [13]. CiteSpace identified citation bursts, with parameters set for a timeline analysis from 1994 to 2024, tracking key research trends using pathfinder pruning [14]. These settings facilitated a visual analysis to generate a keyword timeline map for “total colonic aganglionosis,” offering insights into the temporal development of key research themes.

Bibliometric impact was assessed using h-index, g-index, and m-index from WoSCC [15–17]. The h-index evaluates academic contributions and future impact, while the g-index prioritizes highly cited works. The m-index identifies emerging research trends through keyword bursts and citation analysis. Additionally, journal rankings were assessed using Impact Factor (IF) and Journal Citation Reports (JCR) 2023 to determine their influence in TCA research [14].

## Results

### An overview of publication performance

**Fig. 1:**
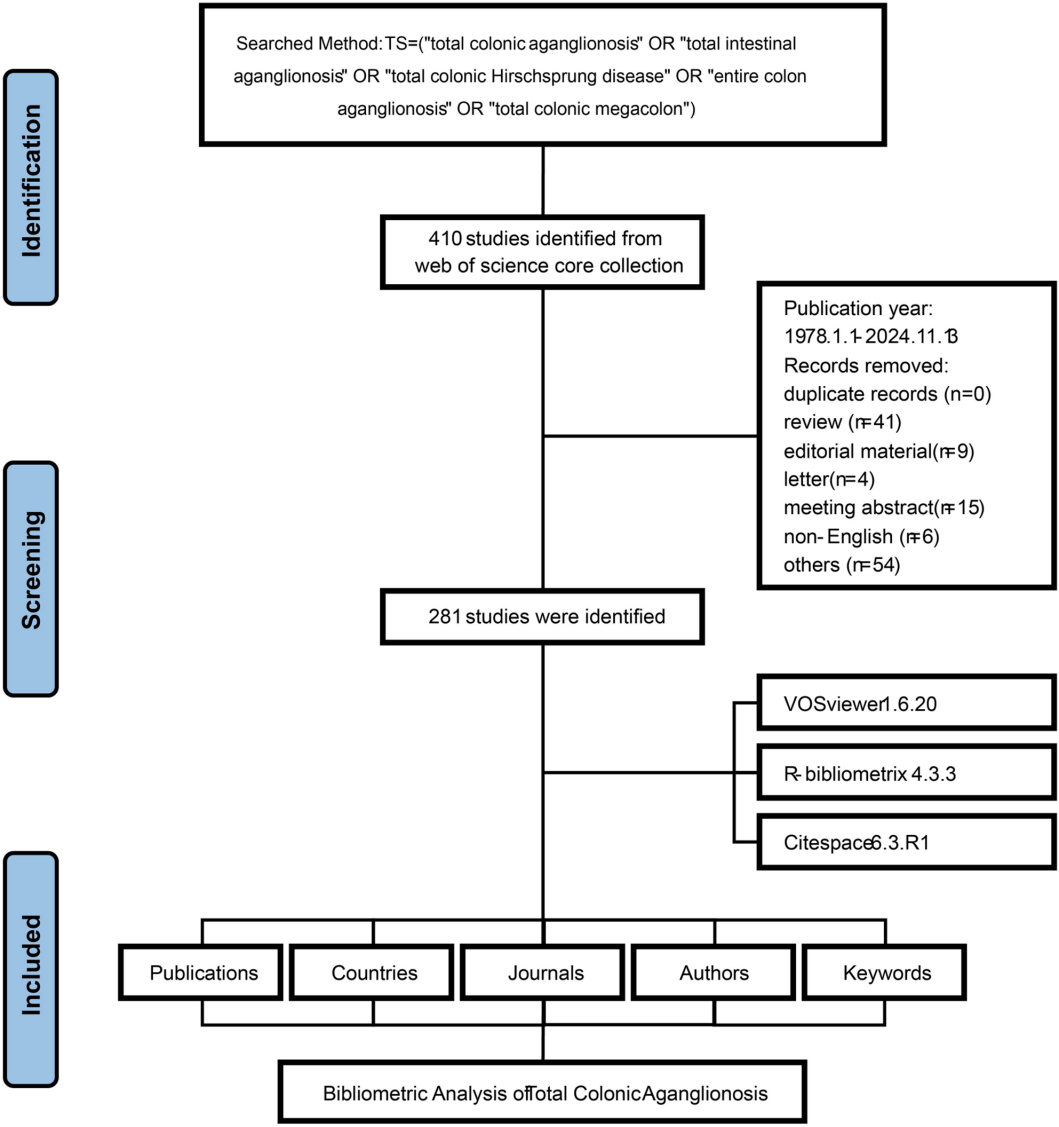
Flowchart of the literature screening process in TCA research

**Fig. 2:**
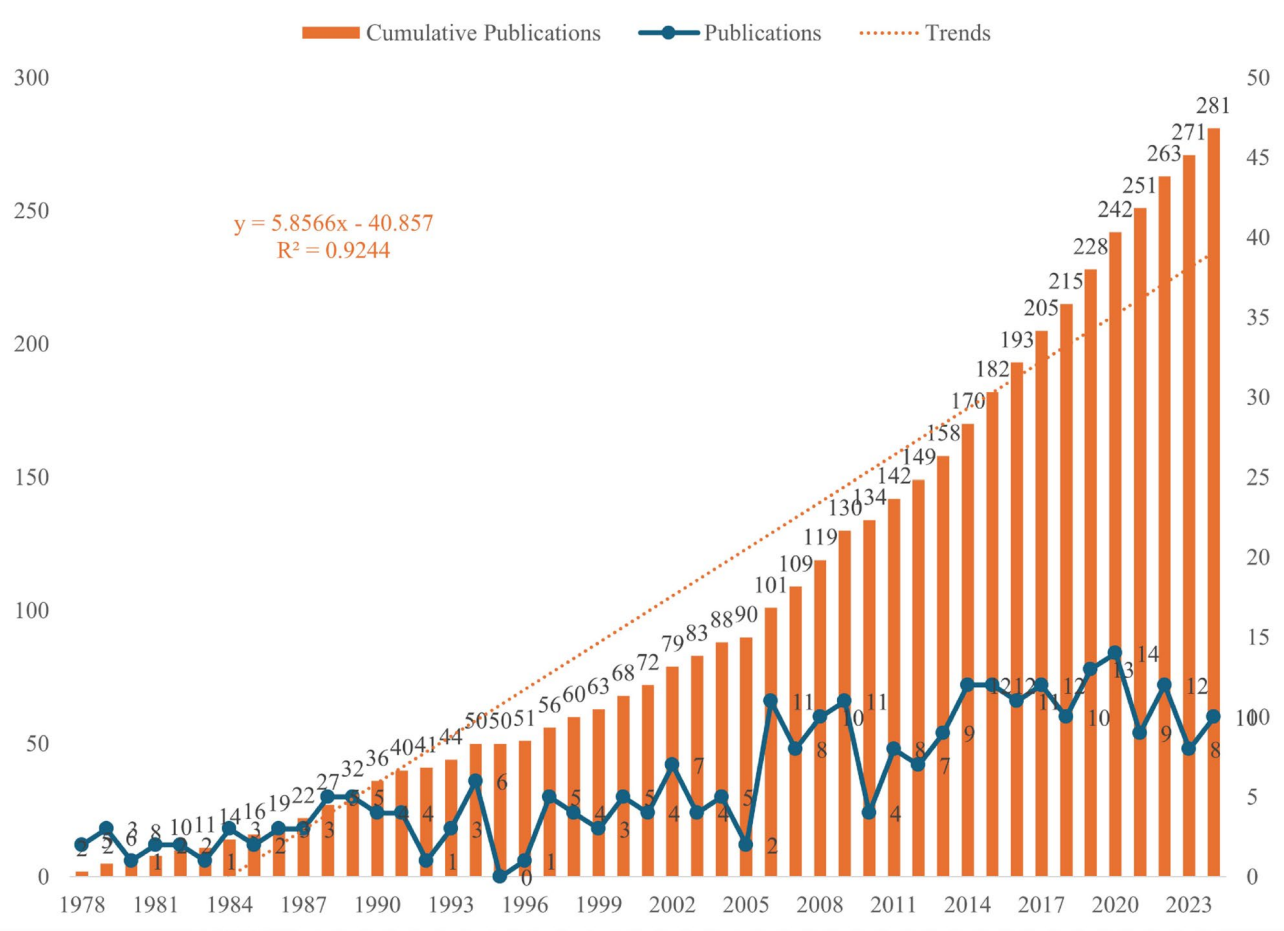
Annual number of publications in TCA research

**Fig. 3:**
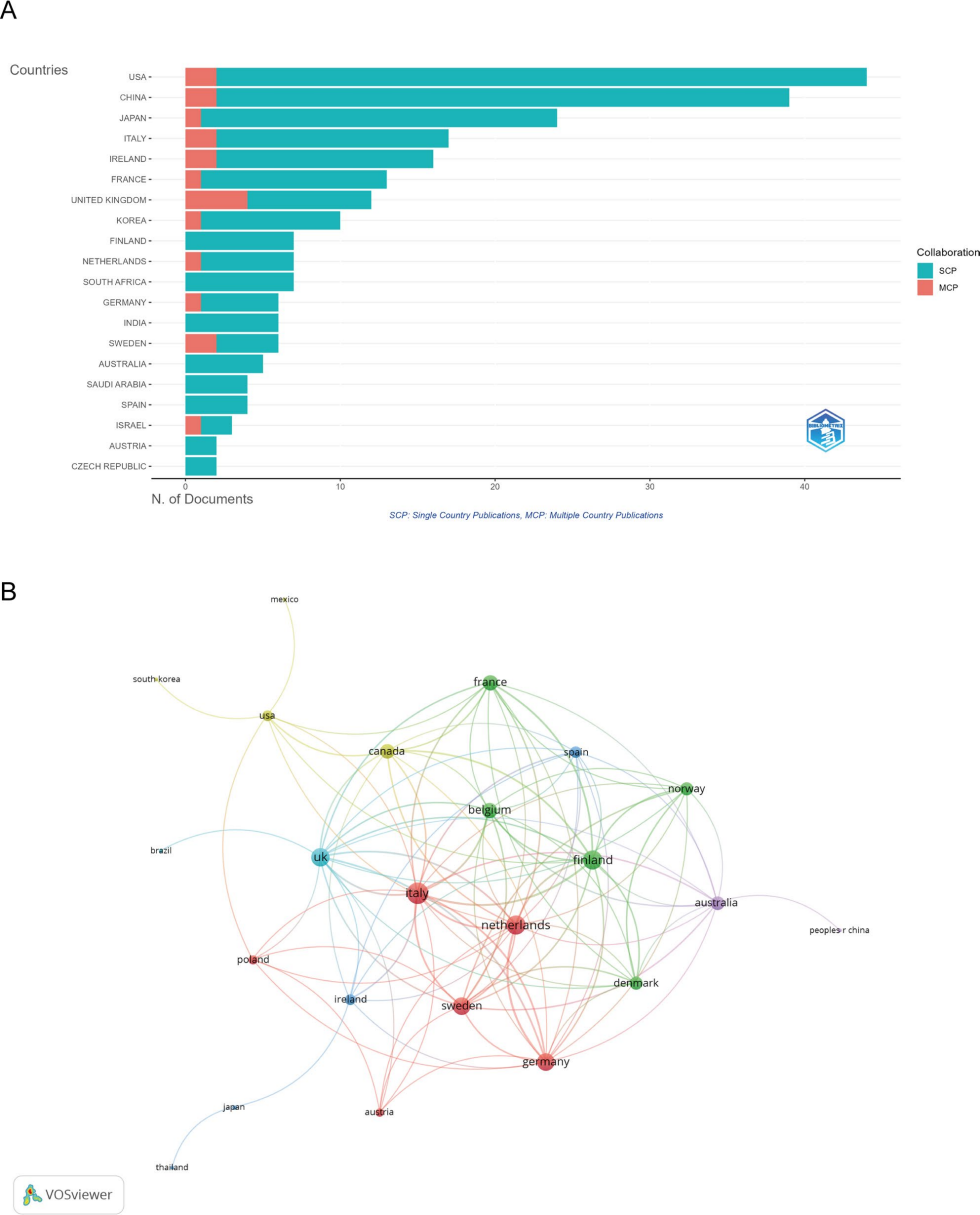
Visualization Map Depicting Country Analysis in TCA Research. (**A**) Distribution of corresponding author’s publications by country (R bibliometrix). SCP: Single Country Publications. MCP: Multiple Country Publications. (**B**) Visualization map depicting the collaboration among different countries

The overall selection process is presented in Fig. 1. The study analyzed 281 papers (1978–2024), involving 1,350 authors and 475 keywords, with 4,010 citations. Research on TCA has steadily increased, averaging 3.56% growth annually (Fig. 2). The top 20 countries by publication volume, primarily in Asia, North America, and Europe, were shown in Fig. 3A and Supplementary Table 1. The USA led in publications (44 papers, 888 citations), followed by China (39 papers, 351 citations) and Japan (24 papers, 293 citations). Finland had the highest average citations per article (50.4), with the USA ranking second (20.2). Collaboration rates were highest in Israel, the UK, and Sweden (33.3%), with Italy, Finland, and the Netherlands forming the strongest international research networks (Fig. 3B).

### Institutional and journal contributions

**Fig. 4:**
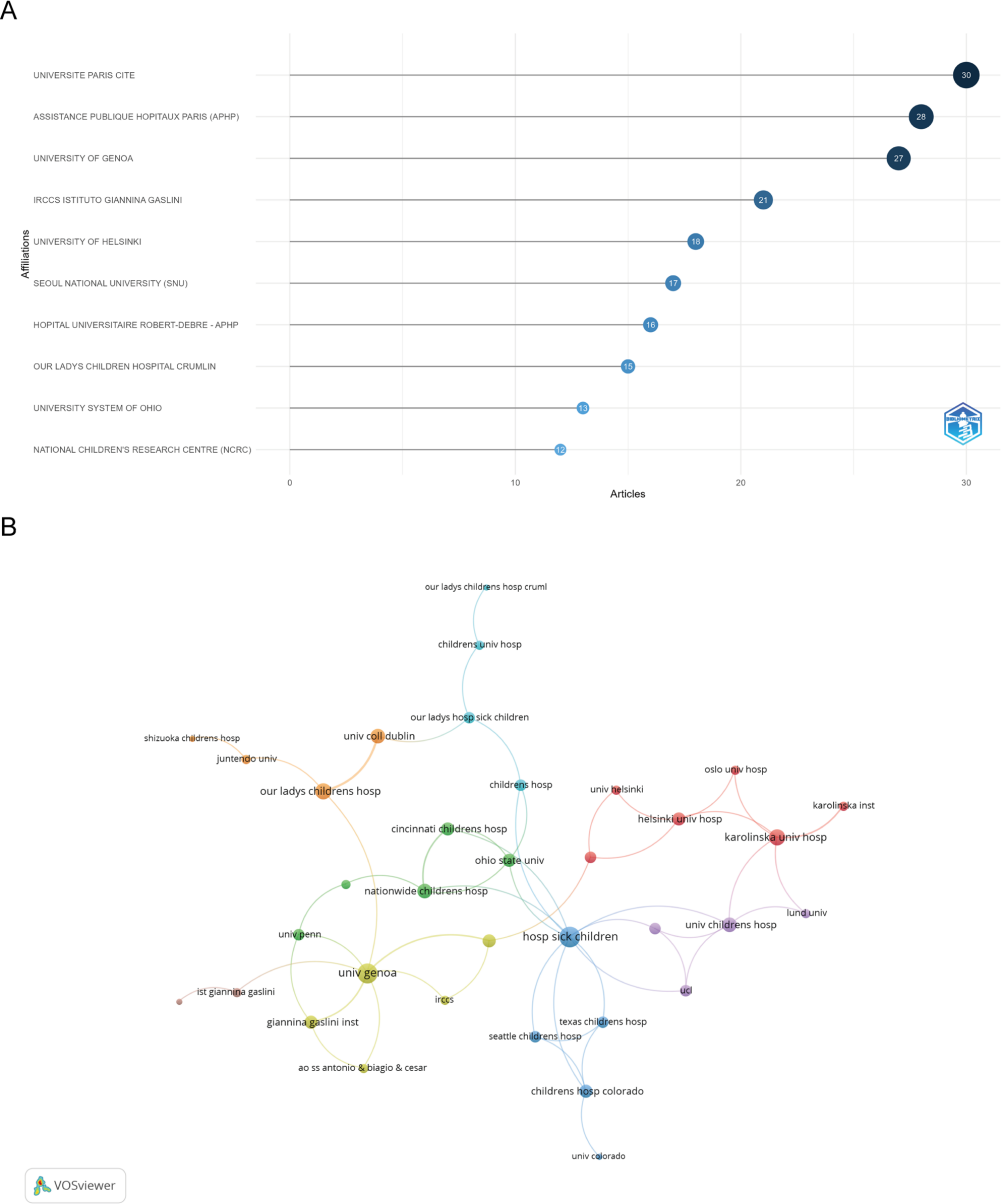
Visualization Map Depicting Institution Analysis in TCA Research. (**A**) Top ten institutions by article count and rank (R bibliometrix). (**B**) Visualization map depicting the collaboration among different institutions

**Fig. 5:**
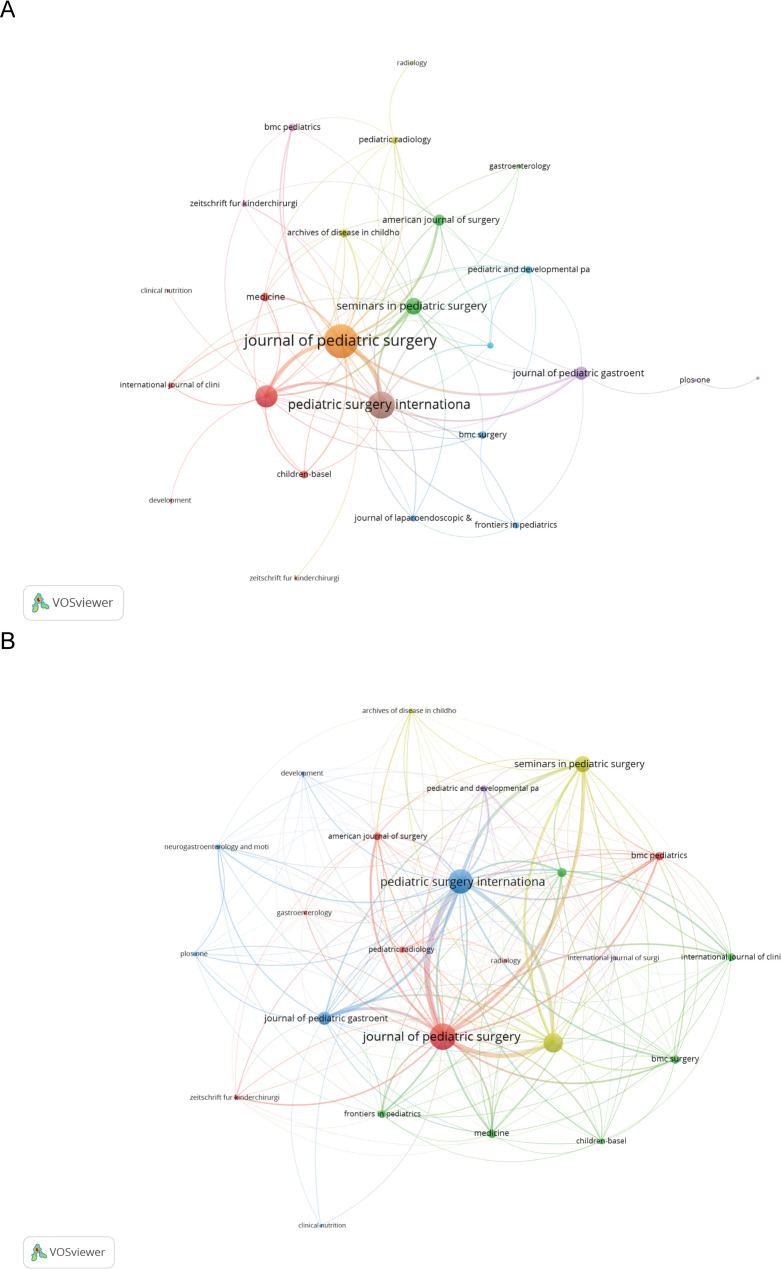
Visualization map depicting the co-occurrence networks of journals and coupling Networks in TCA Research. (**A**) The co-occurrence networks of journals. (**B**) The coupling networks of journals

uropean institutions dominated, with Paris City University (30 papers), Assistance Publique-Hôpitaux de Paris (28 papers), and the University of Genoa (27 papers) leading in publication volume. The Hospital for Sick Children had the highest international collaborations (Fig. 4A & B). In journal rankings, *Journal of Pediatric Surgery* (h-index 22, IF 2.4, 65 papers), *Pediatric Surgery International* (h-index 15, IF 1.5, 59 papers), and *European Journal of Pediatric Surgery* (h-index 12, IF 1.5, 21 papers) were the most productive (Supplementary Table 2). These journals also exhibited the highest link strength in both co-occurrence and coupling networks. *Gastroenterology* (IF 25.7) had the highest impact factor **(**Fig. 5A&B).

### Author contributions and most cited literature

**Fig. 6:**
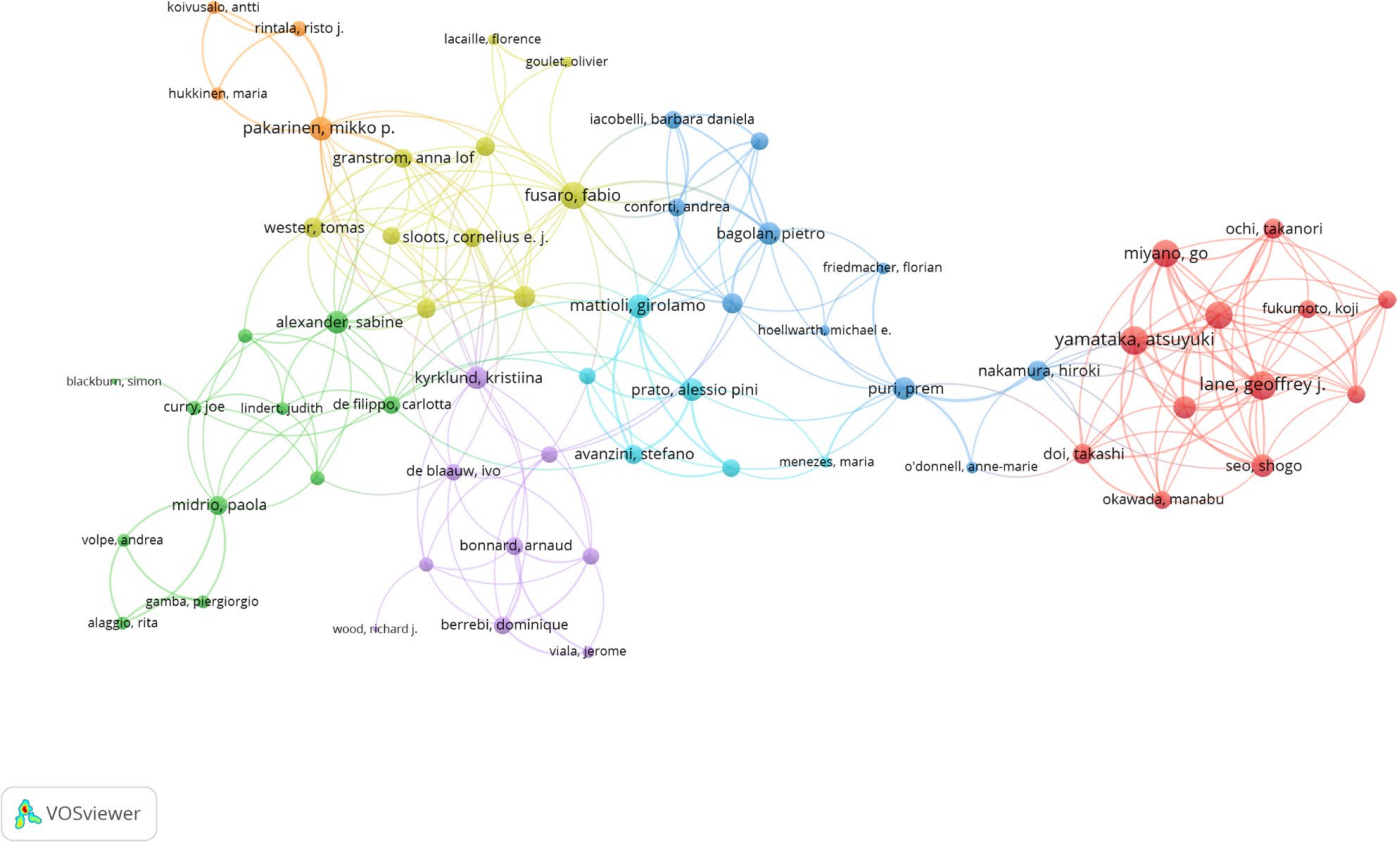
Visualization map depicting the collaboration among different authors in TCA research

Among leading researchers, Prem Puri had the highest h-index (6, 12 papers, 400 citations), followed by Martucciello G., Mattioli Girolamo, Pakarinen Mikko P., and Prato Alessio Pini (h-index 5). Kim Hyun-Young led in international collaborations (Supplementary Tables 3 & Fig. 6).

The most cited paper, published in Nature Genetics (1993, 171 citations), explored genetic mapping in Hirschsprung disease [18]. Other key papers examined RET mutations (Human Mutation, 1997, 114 citations) and long-term bowel function outcomes (Pediatric Surgery International, 2006, 104 citations) [19, 20].

### Analysis of keyword co-occurrence and burst keyword

**Fig. 7:**
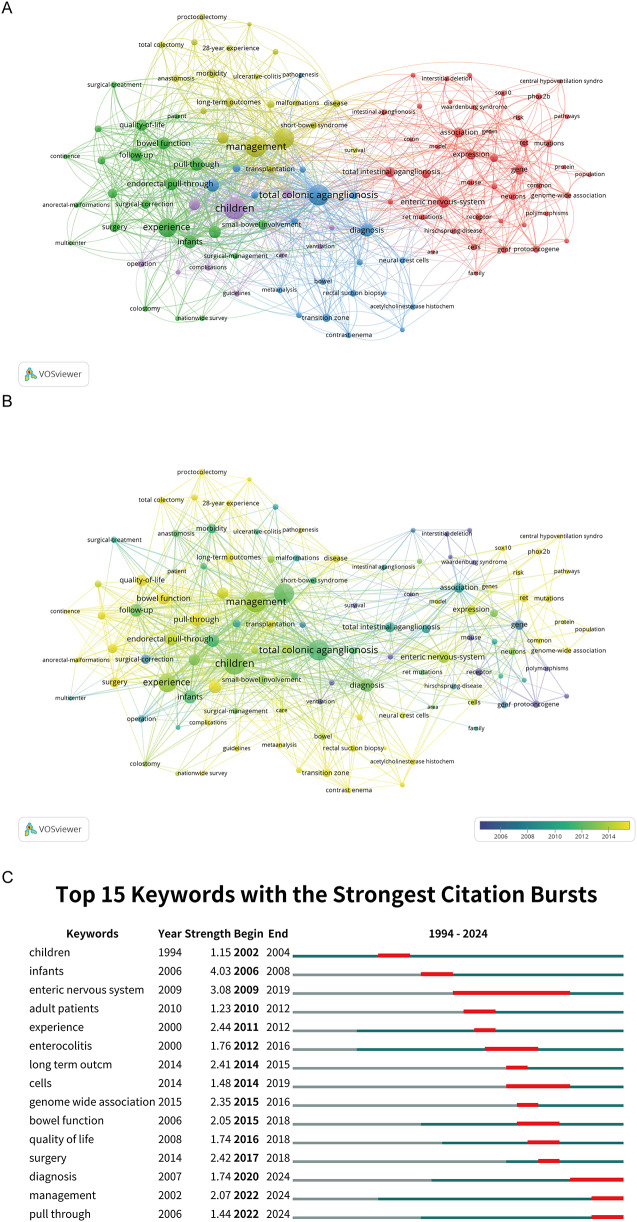
Visualization Map Depicting Keywords in TCA Research. (**A**) Visual analysis of keyword co-occurrence network analysis. (**B**) Visual Analysis of Keyword Co-occurrence Network and Timeline Change Analysis. (**C**) Top 20 Keywords with the Strongest Citation Bursts (CiteSpace)

117 recurring keywords were identified. Cluster 1 (red) explored genetic mutations in TCA, with terms like “mutations”, “gene”, “risk”, and “expression”. Cluster 2 (yellow) focused on management for TCA, including “management”, “long-term outcomes”, and “survival”. Cluster 3 (purple) emphasized clinical guidelines for TCA, featuring keywords such as “guidelines”, “care”, and “children”. Cluster 4 (blue) covered diagnosis for TCA, including “diagnosis”, “rectal suction biopsy”, and “acetylcholinesterase histochemistry”. Cluster 5 (green) highlighted pull-through for TCA, with terms like “pull-through”, “bowel function”, “follow-up”, and “quality-of-life” (Fig. 7A). Chronological analysis revealed early genetic research (pre–2006), clinical management focus (2012), and quality-of-life studies (2014 onward) (Fig. 7B**)**. Recent bursts in citations emphasized “diagnosis” (2020–2024), “management” (2022–2024), and “pull-through” (2022–2024) (Fig. 7C).

## Discussion

### General information

Analysis of 281 publications reveals a clear growth trend in TCA research, reflecting the field’s development. Current hotspots center on genetic mutations, diagnosis, surgical management, and clinical guidelines. Future directions are likely to emphasize precision medicine, genetic discovery, individualized therapies, and functional intestinal recovery.

Geographic analysis shows that TCA research is primarily led by the United States, which has made significant advances in surgical and diagnostic techniques, supported by strong funding and extensive research facilities [9, 11]. China, a rapidly emerging contributor, has increased investment in pathogenesis and genomics, boosting academic productivity in recent years [21, 22]. In Europe, the ERNICA network has proposed clinical guidelines for TCA management [1], with leading institutions in France and Italy contributing substantially to research output and collaboration.

Leading researcher Prem Puri ranks first in both h-index and publication number. Based at University College Dublin, his work focuses on elucidating mechanisms of congenital anomalies including TCA [23, 24]. Journal analysis underscores the central role of pediatric and surgical journals. *Journal of Pediatric Surgery*, *Pediatric Surgery International*, and *European Journal of Pediatric Surgery* are key venues, highlighting the focus on surgical innovation and validation in children with TCA [1, 25, 26]. These journals facilitate communication among researchers translating findings into clinical practice.

### Current hotspots

#### Cluster 1 (Red): Genetic mutations in TCA

A strong association exists between *RET* mutations and TCA. Located on chromosome 10, *RET* encodes a protein essential for enteric nervous system (ENS) development; its mutations lead to defective ganglion formation [27]. Genome-wide association studies (GWAS) have further identified additional implicated genes, including *EDNRB*, *EDN3*, and *SOX10*, which are critical for neural crest and ENS development [28]. While these findings have advanced the understanding of TCA’s genetic basis, the mechanisms underlying variability in prognosis and complications remain unclear [22]. The integration of deep learning and machine learning offers promising avenues for risk prediction, and ongoing research aims to translate genetic insights into tailored therapies, particularly for pediatric patients [22]. These genetic foundations also inform diagnostic approaches and therapeutic strategies discussed in subsequent sections.

#### Cluster 2 (Yellow): Management for TCA

Postoperative management focuses on addressing complications such as short bowel syndrome, chronic nutritional malabsorption, digestive dysfunction, and immune deficiencies, all of which impact long-term outcomes [29]. Early diagnosis and timely intervention are crucial, underscoring the need for systematic follow-up [2]. Multidisciplinary care—incorporating gastroenterology, nutrition, and immunology—is associated with improved outcomes and emphasizes holistic, patient-centered approaches [30]. For cases where surgical repair is not feasible, intestinal transplantation offers an alternative, though it is challenged by risks of graft rejection and infection, requiring long-term immunosuppressive management [31–33]. This comprehensive management approach aligns with clinical guidelines that stress coordinated, multidisciplinary intervention.

#### Cluster 3 (Purple): Clinical guidelines for TCA

Established clinical guidelines emphasize multidisciplinary collaboration in the surgical and perioperative management of TCA, aiding in risk stratification and personalized treatment planning [1]. Common postoperative complications include infection, delayed intestinal recovery, bleeding, and immune-related issues, each affecting recovery and quality of life [1, 25]. Recommendations highlight the importance of prophylactic antibiotics, immunomodulatory therapies when needed, and ventilatory support for high-risk patients [25, 34, 35]. These recommendations provide a structured framework that informs both diagnostic protocols and surgical practices, including the pull-through procedure.

#### Cluster 4 (Blue): diagnosis for TCA

Accurate diagnosis relies on conventional techniques such as rectal suction biopsy and contrast enema. Calretinin immunohistochemistry has now largely replaced acetylcholinesterase histochemistry as the preferred ancillary method [36, 37]. Emerging approaches include advanced imaging (MRI, CT) for prenatal or structural assessment [38], as well as artificial intelligence (AI)-assisted analysis of histological images—leveraging machine learning and deep learning—to enhance diagnostic precision [39]. In addition, genetic testing and genome- GWAS help identify pathogenic mutations and inheritance patterns, thereby supporting molecular diagnosis [40, 41]. These diagnostic advances are closely linked to genetic insights and guide subsequent surgical and management decisions.

#### Cluster 5 (Green): Pull-through for TCA

First described in 1949, the pull-through procedure remains the standard surgical treatment, though the optimal timing in neonates remains debated, with varying recommendations among surgeons [42]. Minimally invasive techniques, including laparoscopy-assisted approaches, show comparable long-term outcomes to open surgery, while robot-assisted methods are emerging but limited in infants [43, 44]. Intraoperative delineation of aganglionic segments is critical; confocal laser endomicroscopy shows promise as a real-time visualization tool, potentially overcoming limitations of frozen-section biopsy [45, 46]. This procedure directly implements insights from genetic and diagnostic research, and its success is supported by the multidisciplinary management outlined in clinical guidelines.

### Emerging trends

Early research, reflected in keywords such as “children” (2002–2004), “infants” (2006–2008), and “ENS” (2009–2019), focused on elucidating the role of ENS in TCA pathogenesis. ENS development relies on precisely regulated neural crest cell processes, and its disruption underpins the variable extent of aganglionosis seen in TCA [47]. Shared genetic pathways, including key genes such as RET, GDNF, TCF4, and ZEB2, further link ENS development and TCA progression [3]. Sustained interest in TCA complications is evidenced by high-frequency keywords like “enterocolitis” (2012–2016), “long term outcome” (2014–2015), and “bowel function” (2015–2018). TCA commonly leads to chronic intestinal failure, requiring long-term parenteral nutrition. Complications such as enterocolitis and intestinal failure-associated liver disease present significant management challenges [29, 48]. Early surgical diversion and multidisciplinary intestinal rehabilitation are recommended to mitigate these risks [29].

Recent citation growth (2020–2024) signals a shift toward refining surgical protocols and diagnostics. The keyword “diagnosis” highlights advances including high-resolution rectal suction biopsy, contrast-enhanced ultrasound, and molecular genetic testing, with AI and machine learning enhancing detection accuracy [5, 38, 49]. Intraoperative mapping remains crucial, and confocal laser endomicroscopy is emerging as a promising real-time alternative to frozen-section biopsy [45, 46]. “Management” underscores a growing emphasis on multidisciplinary, holistic care involving surgical, nutritional, and psychosocial support [25, 50]. Enhanced surgical techniques and systematic follow-up have improved outcomes, though consensus on optimal “pull-through” timing and technique is still evolving [25]. “Pull-through” remains central to TCA treatment, with ongoing refinements aimed at reducing morbidity and improving functional outcomes [51]. The largest series describe JIAA and Duhamel procedures, though the absence of comparative studies precludes definitive recommendations [1]. Future research should focus on defining the best surgical approach and timing.

### Future directions

Future TCA research is poised to integrate AI-aided diagnostics and multi-omics profiling for earlier, precise detection. Genetic studies, including GWAS, will continue to uncover novel mutations, informing targeted therapies such as gene editing with CRISPR-Cas9 [25, 52]. Precision medicine, tailored to individual genotype and phenotype, is expected to optimize therapeutic efficacy [25, 30]. Innovations in minimally invasive surgery, alongside rehabilitative strategies like microbiota modulation and stem cell-based regenerative medicine, will be key to improving long-term functional outcomes and quality of life [53–55]. Multidisciplinary collaboration remains essential to translate these advances into comprehensive, patient-centered clinical practice.

### Strengths and limitations

This study provides a comprehensive bibliometric analysis of TCA, identifying key trends, influential authors, and collaboration networks. Temporal keyword analysis reveals shifts from genetic research to clinical management and long-term outcomes. However, limitations include potential publication bias. First, the bibliometric analysis was conducted using the WoSCC database, which, while comprehensive, may have excluded relevant studies indexed in other databases such as PubMed or Scopus. This could introduce a selection bias and limit the generalizability of the findings. Second, the analysis was restricted to English-language publications, potentially overlooking important contributions in other languages. Finally, while bibliometric methods offer valuable insights, they had limitations in capturing the full scope and nuances of a research field.

## Conclusion

Our bibliometric analysis reveals that current research on TCA predominantly focuses on genetic mutations, management, clinical guidelines, diagnosis, and pull-through for TCA. Future research efforts should prioritize the development of machine learning-based diagnostic tools for early detection, the implementation of individualized treatment strategies guided by genetic profiling, and the refinement and validation of minimally invasive surgical protocols via multicenter, large-sample clinical trials to improve patient outcomes.

## Supplementary Information

Below is the link to the electronic supplementary material.

Supplementary Material 1

## Data Availability

All data generated or analysed during this study are included in this published article.
