# Supplementary material for: Total colonic aganglionosis: a bibliometric analysis of trends and themes (1978–2024)
Source: Orphanet J Rare Dis. 2026 May 16;21:254. doi: 10.1186/s13023-026-04347-w (PMC13390330; doi:10.1186/s13023-026-04347-w)
Supplement: Supplementary file 1 — Supplementary Material 1 [file 13023_2026_4347_MOESM1_ESM.docx]

**Supplementary Table 1 Publication and Citation Profiles of Leading Countries**

| **Country** | **Articles** | **Freq** | **SCP** | **MCP** | **MCP_Ratio** | **TP** | **TP_rank** | **TC** | **TC_rank** | **Average Citations** |
| --- | --- | --- | --- | --- | --- | --- | --- | --- | --- | --- |
| USA | 44 | 0.157 | 42 | 2 | 0.045 | 122 | 1 | 888 | 1 | 20.2 |
| CHINA | 39 | 0.139 | 37 | 2 | 0.051 | 105 | 2 | 351 | 4 | 9 |
| JAPAN | 24 | 0.085 | 23 | 1 | 0.042 | 45 | 5 | 293 | 6 | 12.2 |
| ITALY | 17 | 0.060 | 15 | 2 | 0.118 | 74 | 3 | 237 | 8 | 13.9 |
| IRELAND | 16 | 0.057 | 14 | 2 | 0.125 | 30 | 9 | 508 | 2 | 31.8 |
| FRANCE | 13 | 0.046 | 12 | 1 | 0.077 | 41 | 7 | 280 | 7 | 21.5 |
| UNITED KINGDOM | 12 | 0.043 | 8 | 4 | 0.333 | 31 | 8 | 348 | 5 | 29 |
| KOREA | 10 | 0.036 | 9 | 1 | 0.100 | 58 | 4 | 75 | 13 | 7.5 |
| FINLAND | 7 | 0.025 | 7 | 0 | 0.000 | 21 | 13 | 353 | 3 | 50.4 |
| NETHERLANDS | 7 | 0.025 | 6 | 1 | 0.143 | 42 | 6 | 65 | 14 | 9.3 |
| SOUTH AFRICA | 7 | 0.025 | 7 | 0 | 0.000 | 11 | 16 | 168 | 9 | 24 |
| GERMANY | 6 | 0.021 | 5 | 1 | 0.167 | 26 | 10 | 132 | 10 | 22 |
| INDIA | 6 | 0.021 | 6 | 0 | 0.000 | 11 | 15 | 24 | 22 | 4 |
| SWEDEN | 6 | 0.021 | 4 | 2 | 0.333 | 24 | 11 | 107 | 12 | 17.8 |
| AUSTRALIA | 5 | 0.018 | 5 | 0 | 0.000 | 22 | 12 | 128 | 11 | 25.6 |
| SAUDI ARABIA | 4 | 0.014 | 4 | 0 | 0.000 | 4 | 30 | 60 | 15 | 15 |
| SPAIN | 4 | 0.014 | 4 | 0 | 0.000 | 10 | 17 | 43 | 17 | 10.8 |
| ISRAEL | 3 | 0.011 | 2 | 1 | 0.333 | 4 | 29 | 1 | 32 | 0.3 |
| AUSTRIA | 2 | 0.007 | 2 | 0 | 0.000 | 7 | 19 | 14 | 27 | 7 |
| CZECH REPUBLIC | 2 | 0.007 | 2 | 0 | 0.000 | 5 | 23 | 17 | 26 | 8.5 |

Note(s): Articles: Publications of Corresponding Authors only. Freq: Frequence of Total Publications. MCP_Ratio: Proportion of Multiple Country Publications. TP: Total Publications. TP_rank: Rank of Total Publications. TC: Total Citations. TC_rank: Rank of Total Citations. Average Citations: The average number of citations per publication.

**Supplementary Table 2 Bibliometric Indicators of High-Impact Journals**

| **Journal** | **H_index** | **IF** | **JCR_Quartile** | **PY_start** | **TP** | **TP_rank** | **TC** | **TC_rank** |
| --- | --- | --- | --- | --- | --- | --- | --- | --- |
| JOURNAL OF PEDIATRIC SURGERY | 22 | 2.4 | Q2 | 1979 | 65 | 1 | 1918 | 1 |
| PEDIATRIC SURGERY INTERNATIONAL | 15 | 1.5 | Q3 | 1988 | 59 | 2 | 518 | 2 |
| EUROPEAN JOURNAL OF PEDIATRIC SURGERY | 12 | 1.5 | Q3 | 1991 | 21 | 3 | 169 | 4 |
| JOURNAL OF PEDIATRIC GASTROENTEROLOGY AND NUTRITION | 7 | 2.4 | Q1 | 1989 | 11 | 4 | 126 | 6 |
| SEMINARS IN PEDIATRIC SURGERY | 5 | 1.4 | Q3 | 2010 | 5 | 6 | 162 | 5 |
| PEDIATRIC AND DEVELOPMENTAL PATHOLOGY | 4 | 1.3 | Q3 | 2003 | 6 | 5 | 55 | 21 |
| JOURNAL OF LAPAROENDOSCOPIC & ADVANCED SURGICAL TECHNIQUES | 3 | 1.1 | Q3 | 2016 | 4 | 8 | 25 | 40 |
| NEUROGASTROENTEROLOGY AND MOTILITY | 3 | 3.5 | Q2 | 2015 | 3 | 10 | 29 | 34 |
| AMERICAN JOURNAL OF SURGERY | 2 | 2.7 | Q1 | 1982 | 2 | 11 | 64 | 18 |
| ARCHIVES OF DISEASE IN CHILDHOOD | 2 | 4.3 | Q1 | 1998 | 2 | 12 | 108 | 7 |
| CLINICAL NUTRITION | 2 | 6.6 | Q1 | 2015 | 2 | 15 | 12 | 84 |
| DEVELOPMENT | 2 | 3.7 | Q1 | 2002 | 2 | 16 | 55 | 19 |
| FRONTIERS IN PEDIATRICS | 2 | 2.1 | Q2 | 2019 | 4 | 7 | 9 | 103 |
| GASTROENTEROLOGY | 2 | 25.7 | Q1 | 1984 | 2 | 17 | 82 | 13 |
| INTERNATIONAL JOURNAL OF SURGICAL PATHOLOGY | 2 | 0.9 | Q3 | 2011 | 2 | 19 | 4 | 196 |
| PEDIATRIC RADIOLOGY | 2 | 2.1 | Q2 | 1988 | 2 | 21 | 55 | 22 |
| PLOS ONE | 2 | 2.9 | Q1 | 2011 | 2 | 22 | 47 | 24 |
| RADIOLOGY | 2 | 12.1 | Q1 | 1978 | 2 | 23 | 37 | 30 |
| ZEITSCHRIFT FUR KINDERCHIRURGIE-SURGERY IN INFANCY AND CHILDHOOD | 2 | NA | NA | 1981 | 2 | 25 | 40 | 29 |
| ACTA RADIOLOGICA-DIAGNOSIS | 1 | NA | NA | 1985 | 1 | 27 | 2 | 280 |

Note(s): H_index: The h-index of the journal, which measures both the productivity and citation impact of the publications. IF: Impact Factor, indicating the average number of citations to recent articles published in the journal. JCR_Quartile: The quartile ranking of the journal in the Journal Citation Reports, indicating the journal's ranking relative to others in the same field (Q1: top 25%, Q2: 25%-50%, Q3: 50%-75%, Q4: bottom 25%). TP: Total Publications. TP_rank: Rank of Total Publications. TC: Total Citations. TC_rank: Rank of Total Citations. Average Citations: The average number of citations per publication. PY_start: Publication Year Start, indicating the year the journal started publication.

**Supplementary Table 3 Publication and Citation Profiles of High-Impact Authors**

| **Authors** | **H_index** | **g-index** | **m-index** | **PY_start** | **TP** | **TP_Frac** | **TP_rank** | **TC** | **TC_rank** |
| --- | --- | --- | --- | --- | --- | --- | --- | --- | --- |
| PURI PREM | 9 | 12 | 0.47 | 2006 | 12 | 4.15 | 1 | 400 | 1 |
| MARTUCCIELLO G | 5 | 5 | 0.15 | 1992 | 5 | 0.60 | 7 | 388 | 2 |
| MATTIOLI GIROLAMO | 5 | 5 | 0.29 | 2008 | 5 | 0.53 | 8 | 128 | 11 |
| PAKARINEN MIKKO P. | 5 | 6 | 0.33 | 2010 | 6 | 1.63 | 3 | 266 | 6 |
| PRATO ALESSIO PINI | 5 | 5 | 0.29 | 2008 | 5 | 0.68 | 10 | 183 | 8 |
| FUSARO FABIO | 4 | 5 | 0.24 | 2008 | 5 | 0.41 | 5 | 61 | 22 |
| GOTO S | 4 | 4 | 0.10 | 1984 | 4 | 1.45 | 12 | 94 | 15 |
| KIM HYUN-YOUNG | 4 | 6 | 0.36 | 2014 | 6 | 0.78 | 2 | 52 | 25 |
| LERONE M | 4 | 4 | 0.12 | 1992 | 4 | 0.35 | 16 | 376 | 3 |
| NAKAMURA HIROKI | 4 | 5 | 0.50 | 2017 | 5 | 1.35 | 9 | 36 | 43 |
| PARK KWI-WON | 4 | 4 | 0.36 | 2014 | 4 | 0.43 | 20 | 50 | 31 |
| RINTALA RISTO J. | 4 | 4 | 0.27 | 2010 | 4 | 1.09 | 21 | 159 | 9 |
| ROMEO G | 4 | 4 | 0.12 | 1992 | 4 | 0.35 | 22 | 376 | 3 |
| WESTER TOMAS | 4 | 4 | 0.40 | 2015 | 4 | 1.21 | 24 | 90 | 17 |
| AVANZINI STEFANO | 3 | 3 | 0.18 | 2008 | 3 | 0.35 | 27 | 104 | 13 |
| BAGOLAN PIETRO | 3 | 4 | 0.38 | 2017 | 4 | 0.36 | 11 | 79 | 18 |
| BERREBI DOMINIQUE | 3 | 3 | 0.19 | 2009 | 3 | 0.35 | 28 | 37 | 41 |
| BOLINO A | 3 | 3 | 0.09 | 1993 | 3 | 0.24 | 29 | 302 | 5 |
| BONNARD ARNAUD | 3 | 3 | 0.19 | 2009 | 3 | 0.35 | 30 | 37 | 41 |
| DOI TAKASHI | 3 | 3 | 0.21 | 2011 | 3 | 0.49 | 34 | 23 | 45 |

Note(s): H_index: The h-index of the journal, which measures both the productivity and citation impact of the publications. g_index: The g-index of the journal, which gives more weight to highly-cited articles. m_index: The m-index of the journal, which is the h-index divided by the number of years since the first published paper. TP: Total Publications. TP_rank: Rank of Total Publications. TC: Total Citations. TC_rank: Rank of Total Citations. Average Citations: The average number of citations per publication. PY_start: Publication Year Start, indicating the year the journal started publication.
